# Development and Application of MLB Human Astrovirus Reverse Genetics Clones and Replicons

**DOI:** 10.21769/BioProtoc.5201

**Published:** 2025-02-20

**Authors:** Hashim Ali, David Noyvert, Valeria Lulla

**Affiliations:** Department of Pathology, University of Cambridge, Cambridge, UK

**Keywords:** Human astrovirus, Reverse genetics, Replicon, Virus rescue, Virus passaging, Replication, Replicon assay and immunodetection

## Abstract

Human astroviruses pose a significant public health threat, especially to children, the elderly, and immunocompromised individuals. Nevertheless, these viruses remain largely understudied, with no approved antivirals or vaccines. This protocol focuses on leveraging reverse genetics (RG) and replicon systems to unravel the biology of MLB genotypes, a key group of neurotropic astroviruses. Using reverse genetics and replicon systems, we identified critical genetic deletions linked to viral attenuation and neurotropism, pushing forward vaccine development. We also uncovered novel replication mechanisms involving ER membrane interactions, opening doors to new antiviral targets. Reverse genetics and replicon systems are essential for advancing our understanding of astrovirus biology, identifying virulence factors, and developing effective treatments and vaccines to combat their growing public health impact.

Key features

• Provides a basic understanding of the molecular biology of MLB astroviruses, aiding in addressing open questions related to virus evolution, replication, and pathogenesis.

• Facilitates the development of novel therapeutics and vaccines.

• Enables rapid testing of antiviral drugs against MLB astroviruses.

## Graphical overview


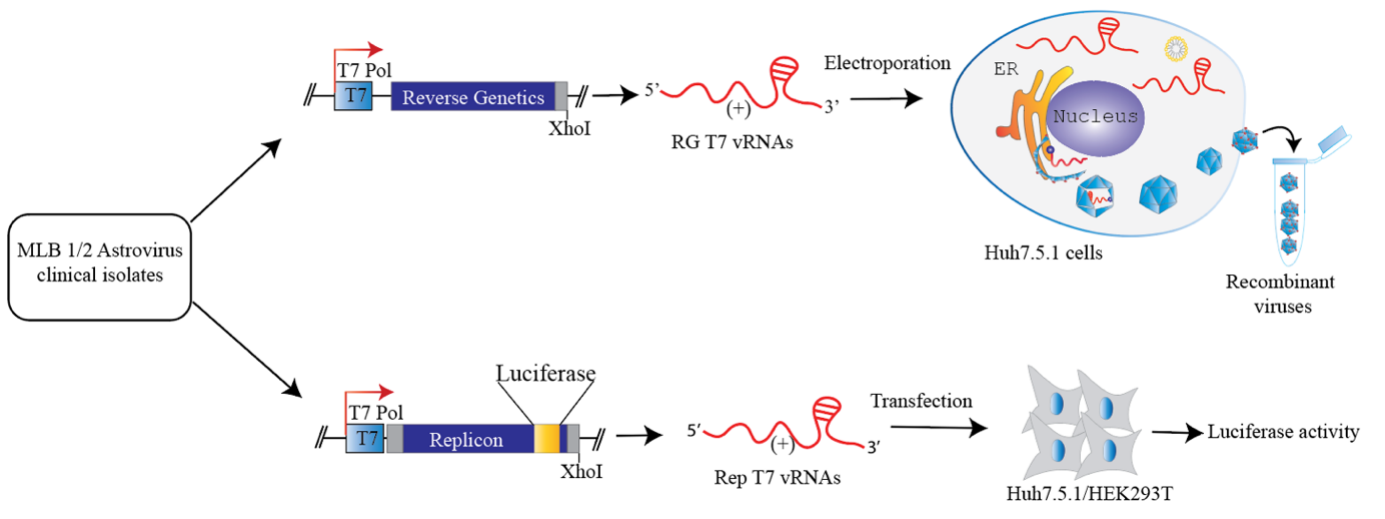


## Background

Human astroviruses (HAstVs) are small, positive-sense RNA viruses primarily associated with gastroenteritis, causing symptoms such as diarrhea, vomiting, and abdominal cramps. These viruses significantly impact public health, particularly affecting young children, the elderly, and immunocompromised individuals, accounting for up to 9% of pediatric gastroenteritis cases globally. There are three major groups of human astroviruses: classical HAstVs, newly emerging HAstV-MLB (Melbourne), and HAstV-VA/HMO (Virginia/human-mink-ovine-like) astroviruses. These viruses are globally prevalent, infect a wide range of species, and demonstrate the capacity for rapid evolution and adaptation to different hosts. Despite their medical importance, many human astroviruses remain understudied due to the lack of essential molecular tools, such as reverse genetics (RG) infectious clones and replicon systems. In addition to causing gastrointestinal disease, certain astroviruses, particularly the MLB genogroup, have been implicated in severe neurological complications, such as encephalitis and meningitis. The MLB genogroup includes distinct viral strains: MLB1, MLB2, and MLB3. Among these, MLB1 is the most prevalent genotype, followed by MLB2 and MLB3. All MLB stains share similarities, including genomic organization and neurotropic potential. Recent studies [1] have developed critical molecular biology tools, such as reverse genetics systems, replicon systems, and immunodetection assays, to investigate the key stages of MLB astrovirus infection. Additionally, novel replication mechanisms were recently uncovered using the MLB RG and replicon systems [2]. These findings revealed interactions between endoplasmic reticulum (ER) membranes and replication factories located in close proximity to the nuclear periphery in a nsP1-dependent manner, providing new insights and potential targets for antiviral therapies.

## Materials and reagents

**Biological materials**

1. Huh7.5.1 cells (obtained from Apath, Brooklyn, New York, United States of America)

2. MLB1 RG infectious virus stocks (from this study, store at -70 °C)

3. MLB2 RG infectious virus stocks (from this study, store at -70 °C)

4. MLB1 replicon based on the reverse genetics of MLB1 (from this study, -20 °C)

5. MLB2 replicon based on the reverse genetics of MLB2 (from this study, -20 °C)

**Reagents**

1. Dulbecco’s modified Eagle’s medium (DMEM) (PAN Biotech, catalog number: P04-03600)

2. Fetal bovine serum (FBS) (PAN Biotech, catalog number: P40-37500)

3. Penicillin-streptomycin (10,000 U/mL) (Life Technologies, catalog number: 15140-122)

4. 200 mM L-Glutamine (Gibco, catalog number: 25030081)

5. Phosphate buffered saline (PBS) (Life Technologies, catalog number: 14190-144)

6. 1 M HEPES buffer, pH range 7.2–7.5 (Life Technologies, catalog number: 15630-080)

7. 0.25% Trypsin-EDTA (Gibco, catalog number: 25200056)

8. Bovine serum albumin (BSA) (PAN Biotech, catalog number: P06-1395500)

9. Direct-zol^TM^ RNA MiniPrep Plus (Zymo Research, catalog number: R2072)

10. Phusion^TM^ Plus DNA polymerase (Thermo Fisher Scientific, catalog number: F630S)

11. XhoI (Thermo Fisher Scientific, catalog number: FD0695)

12. OptiMEM I (Gibco, catalog number: 31985070)

13. RNA Clean-Up and Concentrator kit (Zymo Research, catalog number: R1016)

14. RNaseOUT (Invitrogen, catalog number: 10777019)

15. Paraformaldehyde solution (PFA), 4% in PBS (Thermo Fisher Scientific, catalog number: 30525-89-4)

16. Βeta-mercaptoethanol (Sigma, catalog number: M3148)

17. T7 mMESSAGE mMACHINE Transcription kit (Thermo Fisher Scientific, catalog number: AM1344)

18. T7 Firefly luciferase plasmid (Promega, catalog number: L482A)

19. QIAquick Gel Extraction kit (Qiagen, catalog number: 28704)

20. QIAquick PCR Purification Kit (Qiagen, catalog number: 28104)

21. Dual-Luciferase Reporter Assay System (Promega, catalog number: E1980)

22. Anti-CP antibody (custom-made rabbit polyclonal antibody)

23. Anti-tubulin antibody (Abcam, mouse monoclonal antibody, catalog number: ab6160)

24. IRDye 800 CW goat anti-rabbit secondary antibody (LI-COR Biotechnology, catalog number: 926-32211)

25. IRDye 680 RD goat anti-mouse IgG secondary antibody (LI-COR Biotechnology, catalog number: 926-68070)

26. Anti-dsRNA antibody (anti-dsRNA IgG2a) (Scicons J2, catalog number: 10010500)

27. Goat anti-rabbit IgG (H+L) secondary antibody Alexa 488-conjugated (Thermo Fisher Scientific, catalog number: A-11008)

28. Goat anti-mouse IgG (H+L) secondary antibody 594-conjugated (Thermo Fisher Scientific, catalog number: A-11005)

29. Nitrocellulose membrane, 0.2 μm (Thermo Fisher Scientific, catalog number: 15209874)

30. 8% Sodium azide solution (Thistle Scientific, catalog number: SBL-40-2008-01)

31. MEM non-essential amino acid solution (Sigma, catalog number: M7145)

32. Gibco^TM^ VP-SFM media (Fisher Scientific, catalog number: 10593273)

33. Normal goat serum (Abcam, catalog number: ab7481)

34. DNase I (Zymo Research, catalog number: E1011)

35. Lipofectamine 2000 (Thermo Fisher Scientific, catalog number: 11668019)

36. QIAGEN Plasmid Plus Midi Sample kit (Qiagen, catalog number: 12941)

37. Agarose (Sigma-Aldrich, catalog number: A9539)

38. Tween-20 (Sigma-Aldrich, catalog number: P1379)

39. PIPES (Sigma-Aldrich, catalog number: P6757)

40. EGTA (Sigma-Aldrich, catalog number: 03777)

41. MgCl_2_ (Sigma-Aldrich, catalog number: 31413)

42. NaOH (Sigma-Aldrich, catalog number: 06203)

43. Glycerol (Sigma-Aldrich, catalog number: G5516)

44. Triton X-100 (Thermo Fisher Scientific, catalog number: HFH10)

45. Tris HCl (Duchefa Biochemie, catalog number: T1513)

46. SDS (Sigma-Aldrich, catalog number: 75746)

47. pAVIC plasmid [3]

48. Bromophenol blue (Sigma-Aldrich, catalog number: B0126)

49. Dried skimmed milk powder (Marvel, multiple suppliers, grocery stores)

50. Formaldehyde, methanol free (Polysciences, catalog number: 04018-1)

51. Hoechst stain (Thermo Fisher Scientific, catalog number: 62249)

52. Wheat germ agglutinin (WGA) (Thermo Fisher Scientific, catalog number: W11261)

53. Perasafe (Medisave, catalog number: UN068)

54. Chemgene (Thermo Fisher Scientific, catalog number: SKU075A)

55. Qiagen AVL lysis buffer (Qiagen, catalog number: 19073)

56. Zymo-Spin IC columns (Zymo Research, catalog number: C1004-50)

57. Invitrogen SuperScript III Reverse Transcriptase (Thermo Fisher Scientific, catalog number: 18080044)

58. RNase-free water (Qiagen, catalog number: 129112)

59. 10% Chloros (sodium hypochlorite) (Thermo Fisher Scientific, catalog number: 219255000)

**Solutions**

1. Complete media (DMEM-GHAA-10% FBS) (see Recipes)

2. DMEM-GHAA-5% FBS media (see Recipes)

3. Infection media (DMEM-GHAA+0.2% BSA) (see Recipes)

4. DMEM-GH-2% FBS media (see Recipes)

5. DMEM-GH media (see Recipes)

6. Transfection media (15 mL)

7. Serum-free media (DMEM-GHAA) (see Recipes)

8. VPSF-GHAA media (see Recipes)

9. Antibody solution (see Recipes)

10. 2× PHEM buffer (see Recipes)

11. 2× Loading buffer + beta mercaptoethanol (2× LB-βME) (see Recipes)

12. 0.08% Trypsin-EDTA solution (see Recipes)

13. 1% Perasafe solution (see Recipes)

14. 5% Chemgene solution (see Recipes)

15. 0.1% PBST solution (see Recipes)

**Recipes**

**1. Complete media (DMEM-GHAA-10% FBS) (500 mL)**

**Table:** 

Reagent	Final concentration	Quantity or Volume
DMEM	n/a	429 mL
100% FBS	10%	50 mL
1 M HEPES	20 mM	10 mL
Penicillin and streptomycin	20 Units/mL	1 mL
100× MEM non-essential amino acids	n/a	5 mL
200 mM L-glutamine	2 mM	5 mL
Total (optional)	n/a	500 mL

**2. DMEM-GHAA-5% FBS media (500 mL)**

**Table:** 

Reagent	Final concentration	Quantity or Volume
DMEM	n/a	454 mL
100% FBS	5%	25 mL
200 mM L-glutamine	2 mM	5 mL
1 M HEPES	20 mM	10 mL
Penicillin and streptomycin	20 Units/mL	1 mL
100× MEM non-essential amino acid	n/a	5 mL
Total (optional)		500 mL

**3. Infection media (DMEM-GHAA+0.2% BSA) (50 mL)**

**Table:** 

Reagent	Final concentration	Quantity or Volume
DMEM	n/a	46.9 mL
200 mM L-glutamine	2 mM	0.5 mL
1 M HEPES	20 mM	1 mL
Penicillin and streptomycin	20 Units/mL	0.1 mL
100× MEM non-essential amino acid	n/a	0.5 mL
10% BSA	0.2%	1.0 mL
Total (optional)	n/a	50 mL

**4. DMEM-GH-2% FBS media (500 mL)**

**Table:** 

Reagent	Final concentration	Quantity or Volume
DMEM	n/a	475 mL
100% FBS	2%	10 mL
1 M HEPES	20 mM	10 mL
200 mM L-glutamine	2 mM	5 mL
Total (optional)	n/a	500 mL

**5. DMEM-GH media (500 mL)**

**Table:** 

Reagent	Final concentration	Quantity or Volume
DMEM	n/a	485 mL
200 mM L-glutamine	2 mM	5 mL
1 M HEPES	20 mM	10 mL
Total (optional)	n/a	500 mL

**6. Transfection media (15 mL)**

**Table:** 

Reagent	Final concentration	Quantity or Volume
OptiMEM	n/a	15 mL
RNaseOUT	40 Units/mL	15 μL
Total (optional)	n/a	15 mL

**7. Serum-free media (DMEM-GHAA) (500 mL)**

**Table:** 

Reagent	Final concentration	Quantity or Volume
DMEM	n/a	479 mL
1 M HEPES	20 mM	10 mL
Penicillin and streptomycin	20 Units/mL	1 mL
100× MEM non-essential amino acids	n/a	5 mL
200 mM L-glutamine	2 mM	5 mL
Total (optional)	n/a	500 mL

**8. VPSF-GHAA media (50 mL)**

**Table:** 

Reagent	Final concentration	Quantity or Volume
VP-SFM media	n/a	47.9 mL
1 M HEPES	20 mM	1 mL
Penicillin and streptomycin	20 Units/mL	0.1 mL
100× MEM non-essential amino acids	n/a	0.5 mL
200 mM L-glutamine	2 mM	0.5 mL
Total (optional)	n/a	50 mL

**9. Antibody solution (100 mL)**

**Table:** 

Reagent	Final concentration	Quantity or Volume
PBS	1×	100 mL
100% goat serum	0.5%	0.5 mL
8% sodium azide	0.02%	0.25 mL
Total (optional)	n/a	100 mL

**10. 2× PHEM buffer (500 mL)**

**Table:** 

Reagent	Final concentration	Quantity or Volume
PIPES	120 mM	72.56 g
HEPES	50 mM	23.84 g
EGTA	20 mM	15.2 g
2 M MgCl_2_	4 mM	1 mL
H_2_O	n/a	see note*
Total (optional)	n/a	500 mL

*First dissolve PIPES in 400 mL of water and then add the other reagents. Adjust pH to 7.2 with 5 M NaOH or KOH.

**11. 2× loading buffer + beta-mercaptoethanol (2× LB-βME) (10 mL)**

**Table:** 

Reagent	Final concentration	Quantity or Volume
1 M Tris HCl pH 6.8	125 mM	1.25 mL
10% SDS	4%	4 mL
1% Bromophenol blue	0.04%	0.4 mL
100% glycerol	20%	2 mL
Beta-mercaptoethanol	10%	1 mL

Then, add water to make a final volume of 10 mL.

**12. 0.08% Trypsin-EDTA solution**

Add 2.5 mL of 0.25% trypsin-EDTA in 7.5 mL of PBS.

**13. 1% Perasafe solution**

1 g of Perasafe powder in 100 mL of water.

**14. 5% Chemgene solution**

Add 50 mL of Chemgene in 1,000 mL of water.

**15. 0.1% PBST solution**

Add 1 mL of Tween-20 in 1,000 mL of PBS.

**Laboratory supplies**

1. Pipette tips (Thermo Fisher Scientific, catalog numbers: 02-707-426, 02-707-403, 02-707-438)

2. 1.5 mL microfuge tubes (STARLAB, catalog number: 51615-5500)

3. 15 mL Falcon tubes (Corning, catalog number: 430052)

4. 50 mL Falcon tubes (Corning, catalog number: 430829)

5. T175 tissue culture flasks (TPP^®^ tissue culture flasks) (Sigma, catalog number: Z707562)

6. 6-multiwell polystyrene culture plates (TPP^®^ tissue culture plates) (Sigma, catalog number: Z707767)

7. 12-multiwell polystyrene culture plates (TPP^®^ tissue culture plates) (Sigma, catalog number: Z707775)

8. IBIDI 8-well chambered slides (Ibidi GmbH, catalog number: 80807-90)

9. 96-well flat-bottom plate (TPP, catalog number: Z707902)

10. U-bottom 96-well plate (TPP, catalog number: Z707899)

11. 0.2 μM filters (Appleton, catalog number: ACF141)

12. Screw-cap tubes (Axigen, catalog number: SCT-150-A-S)

## Equipment

1. Biosafety level 2 culture cabinet (Wolflabs, model: BioMAT 2 BM21800R)

2. -70 °C freezer (Eppendorf, catalog number: F660320001)

3. Vortex (Heidolph, model: Reax Top)

4. Spray bottle with 70% ethanol (Sigma-Aldrich, catalog number: 32221)

5. Pipettes: 1,000 μL (StarLab, catalog number: S1111-6001)

6. Pipettes: 200 μL (StarLab, catalog number: S1111-0000)

7. Pipettes: 10 μL (StarLab, catalog number: S1111-3000)

8. Pipettes: 2 μL (StarLab, catalog number: S1111-3000)

9. 25 mL pipettes (Corning, catalog number: 760 180)

10. 10 mL pipettes (Corning, catalog number: 4488)

11. CO_2_ incubator (PHCBI, catalog number: MCO-230AIC)

12. Hemocytometer (Logos BioSystems, catalog number: L40002)

13. Cell counting slides (Logos BioSystems, catalog number: L12002)

14. Rocking platform shaker (Cole-Parmer^TM^, catalog number: WZ-51900-30)

15. Phase-contrast inverted microscope (Nikon TMS Inverted Microscope)

16. 4 °C refrigerator (LabCold, catalog number: RLPR0517)

17. Autoclave (BMM Weston, custom-made)

18. Bio-Rad Gene Pulser Xcell system (Bio-Rad, catalog number: 1652661)

19. Electroporation cuvettes (Bio-Rad, catalog number: 1652091)

20. Water bath (Clifton, catalog number: ZT1250337S)

21. LI-COR ODYSSEY CLx imager (LicorBio, catalog number: 9140-09)

22. Leica SP5 confocal microscope (Leica Microsystems, catalog number: Leica DMI6000 CS)

23. Trans-Blot Turbo transfer system (Bio-Rad, catalog number: 1704150EDU)

24. SDS-PAGE running assembly (Bio-Rad, catalog number: 1658001FC)

25. GloMax^®^ Navigator microplate luminometer (Promega, catalog number: GM2010)

26. NanoDrop^TM^ 2000 spectrophotometer (Thermo Fisher Scientific, catalog number: ND2000)

## Software and datasets

1. ImageJ 1.47v (10.2)

2. Prism v8.2.2 (GraphPad, July 2019)

## Procedure

**CRITICAL:** All experiments with MLB RG viruses (i.e., all pre-fixation and pre-lysis steps) should be performed inside a biosafety level 2 (BSL2/CL2) tissue culture laboratory according to the country and institution regulations and required permits regarding handling and storage of human astroviruses.

**Part I: Production of infectious recombinant MLB1 and MLB2 astrovirus stocks**

**A. Engineering MLB reverse genetics**

1. To construct RG clones for MLB1 and MLB2, specific primers are designed using the 5′ and 3′-terminal consensus sequences (Table 1).

**Table 1.** List of primers

	Primers	Sequences (5′ to 3′)
1	MLB1 forward	GAGTAATACGACTCACTATAGCCAAGAGTGGTGGTATGGCTG
2	MLB1 reverse	CCATACATTTATGCTGGAAGAAAAAAAGC
3	MLB2 forward	GAGTAATACGACTCACTATAGCCAAGAGTGGTAGGATGGCTGTG
4	MLB2 reverse	CCTCTAAATCTACCTGATTAGAAAAAAAAAGATAAAATTTTATTTGTC

2. The viral genomic RNAs are extracted from clinical isolates (GenBank accession number MLB1-MK089434 and MLB2-MK089435) using Qiagen AVL lysis buffer and Zymo spin RNA purification columns.

3. Elute viral genomic RNAs in 30 μL of RNase-free water and quantify using a Nanodrop.

4. For cDNA synthesis, use 11 μL of viral RNA (25–100 ng) using Superscript III reverse transcriptase (Table 2).

**Table 2.** cDNA synthesis reaction mixture 1

	Component	Volume (μL)
1	viral RNA	11 μL (25 ng to 100 ng)
2	dNTPs	1.0 μL (10 mM)
3	Virus-specific reverse primer	1.0 μL (100 μM)
Total volume		13 μL

5. Incubate room temperature (RT) reaction at 65 °C for 5 min and cool down to 4 °C.

6. Add 7 μL of reverse transcriptase mix to each PCR reaction (4 μL of 5× first strand buffer + 1 μL of 100 mM DTT (Dithiothreitol) + 1 μL of RNaseOUT +1 μL of SuperScript III Reverse Transcriptase enzyme).

7. Incubate at 55 °C for 60 min, followed by incubation at 70 °C for 15 min and at 10 °C on hold.

8. Full-length genomes of MLB1 and MLB2 are amplified by PCR using Phusion High-Fidelity DNA polymerase and virus-specific primer sets (Table 3).

**Table 3.** PCR amplification reaction

	Component	Volume (μL)
1	cDNA (template), 25 ng to 100 ng	1.0 μL
2	5× HF buffer	10.0 μL
3	10 mM dNTPs	1.0 μL
4	Virus-specific forward primer, 100 μM	0.3 μL
5	Virus-specific reverse primer, 100 μM	0.3 μL
6	Phusion DNA polymerase	0.5 μL
7	DMSO	1.0 μL
8	Water	36.0 μL
Total volume		50 μL

**Figure 1.**
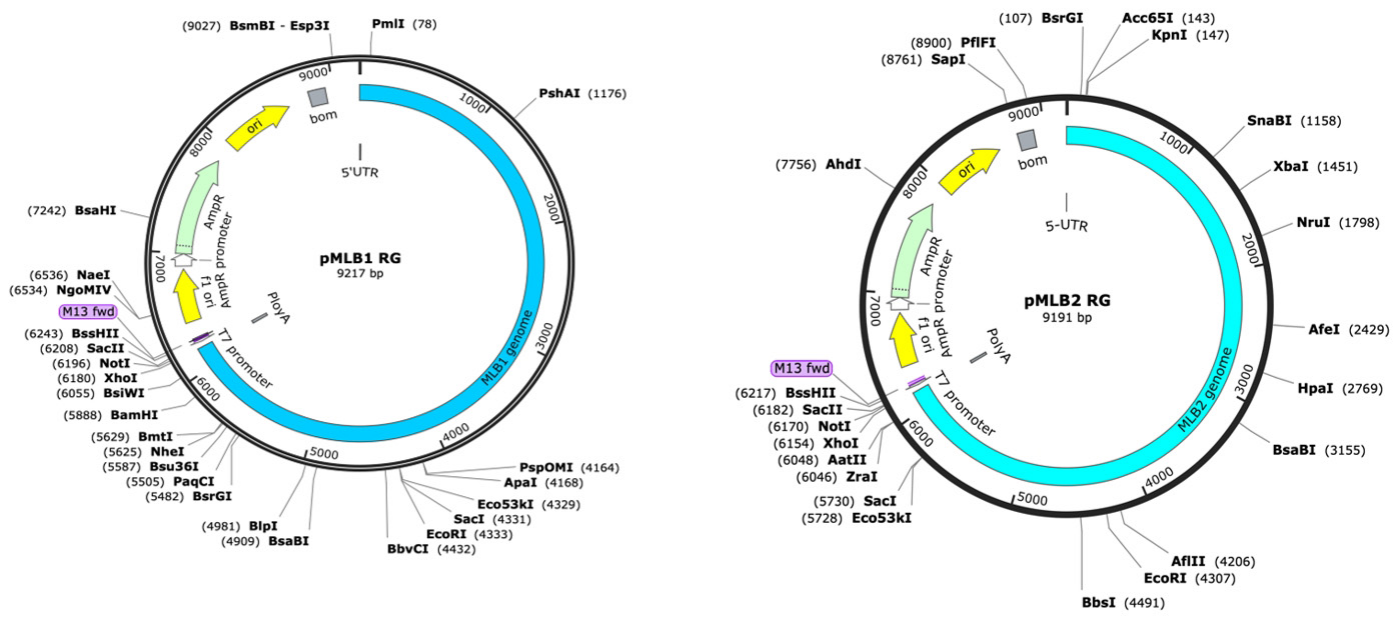
Plasmid maps of reverse genetics (RG) clones of MLB1 and MLB2, generated using SnapGene. The map on the left represents MLB1, while the map on the right represents MLB2.

9. Perform PCR using the following steps: (step 1) 98 °C for 1 min; (step 2, 35 cycles): 98 °C for 10 s, 57 °C for 10 s, 72 °C for 3 min; (step 3): 72 °C for 5 min; and hold at 10 °C.

10. Separate amplified viral genomes on 0.7% agarose gel, purify using Zymo column, and sequence with specific primers.

11. The amplified viral genomes of MLB1 and MLB2 are cloned into the plasmid pAVIC [3] by replacing HAstV1 genome with MLB1 or MLB2 under T7 promoter using a single-step ligation-independent cloning method.

12. See plasmid maps of reverse genetics clones of MLB1 and MLB2 in Figure 1.

13. RG plasmids were transformed into XL1Blue competent cells using a standard protocol. Single positive colonies were selected to further amplify bacterial cultures, followed by plasmid purification using QIAGEN Plasmid Plus Midi kit.

**B. Linearization and in vitro synthesis of T7 RNA transcripts**

1. To linearize, digest the MLB RG clones with the XhoI restriction enzyme (as shown in Table 4) and their corresponding GNN mutants (RdRp knockout as negative control).

**Table 4.** Restriction digestion reaction

	Component	Volume (μL)
1	Plasmids: MLB1/MLB2/GNN (1 μg/μL)	5.0 μL
2	XhoI (10 U/μL)	1.0 μL
3	10× FD buffer	5.0 μL
4	MilliQ water	39.0 μL
Total volume		50 μL

2. Incubate digestion reactions at 37 °C either in a water bath or incubator for 2 h.

3. Purify the linearized plasmids using a PCR Clean-Up kit using standard manufacturer’s instructions and elute in 20 μL of nuclease-free water or elution buffer. Quantify the concentrations of the linearized plasmids using a Nanodrop.

4. Synthesize T7 RNA transcripts using the T7 mMESSAGE mMACHINE Transcription kit (as shown in Table 5).

**Table 5.** In vitro synthesis of MLB RG T7 RNA transcripts

	Component	Volumes
1	Linear plasmids (MLB1/MLB2/GNN) (150–400 ng)	3.0 μL
2	2× NTP/CAP	5.0 μL
3	10× transcription buffer	1.0 μL
4	T7 Enzyme mixture	1.0 μL
Total reaction volume		10 μL

5. Incubate the T7 RNA reactions at 37 °C for 1 h (Note: No DNaseI treatment). To evaluate the quality of the T7 RNA samples, check them by loading 0.5 μL of RNA on a 1% agarose gel before electroporation into Huh7.5.1 cells. At this point, the T7 RNA samples can be directly used for electroporation or stored at -70 °C.

**C. Electroporation of T7 RNA transcripts**

1. First, check the Huh7.5.1 cells’ quality and confluency; they should be 80%–90% confluent (T175 flask is enough for 3–4 electroporations in the 6-multiwell plate format) before plating. Using cells from overconfluent flasks would impact the electroporation efficiency of T7-transcribed RNA(s). Prechill the required number of electroporation cuvettes in closed plastic bags (number of samples + mock) at 4 °C to avoid moisture condensation.

2. Prewarm complete media and 0.08% trypsin-EDTA solution at 37 °C.

3. Discard the culture media from the flask and rinse Huh7.5.1 cells with 10 mL of 1× PBS.

4. Add 15 mL of 1× PBS and incubate for at least 3–5 min (to completely remove the FBS-containing media).

5. Add 5 mL of prewarmed 0.08% trypsin-EDTA, seal the flask completely, and then gently rotate the flask to ensure that trypsin is equally distributed to the monolayer of Huh7.5.1 cells. Incubate at room temperature for 2–3 min to trypsinize the cells.

6. Add 10 mL of complete media to neutralize trypsin, resuspend cells properly using a pipette, and collect cells into a 50 mL Falcon tube.

*Note: Cells should not be over-trypsinized as it can reduce electroporation efficiency.*

**Figure 2.**
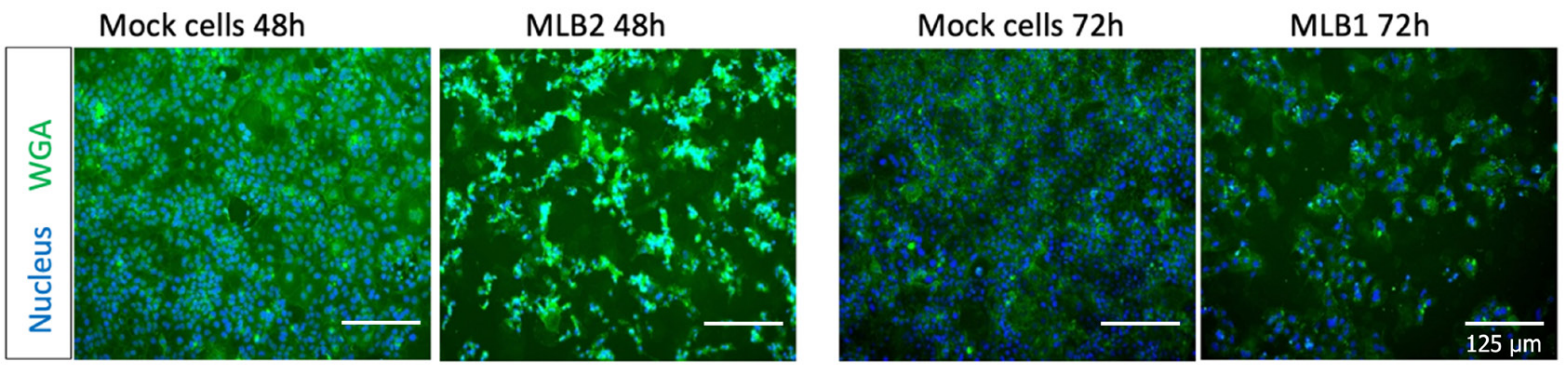
Representative images showing the MLB2 and MLB1 virus-induced CPE in Huh7.5.1 cells

7. Centrifuge cells at 400× *g* for 5 min with no brake (it will take 10–15 min to stop). Discard the supernatant and carefully remove any leftover media using a 1 mL pipette.

8. Add 45 mL of 1× PBS to the cell pellets. If the cells are in more than one Falcon tube, combine them into one Falcon tube for washing. At this point, count the cells using a hemocytometer; ~5 × 10^5^ cells are enough for one electroporation. Take the required number of cells for electroporation and centrifuge the cells at 400× *g* for 5 min with no brake function.

9. Discard PBS and resuspend again cells in PBS (800 μL of PBS per electroporation).

10. Proceed immediately to electroporation using Bio-Rad Gene Pulser Xcell system. Start with mock cell electroporation (use the same volume of 1× transcription buffer), then proceed with viral T7-transcribed RNA(s) from Table 5.

11. Add 750 μL of cells and 10 μL (~20 μg) of T7-transcribed RNA(s) into the cuvette and mix well.

12. Set the electroporator to the exponential protocol, 800 V, 25 μF, 4 mm cuvette. Pulse once; the expected time constant should be 0.3–0.5 ms. Mix the cells in the closed cuvette by tapping, then repeat the pulse with the same expected time constant.

13. Quickly add 500 μL of DMEM-GHAA-5% FBS media to the cuvette, mix, and transfer the cells to 1.5 mL microcentrifuge tubes. The same procedure is to be followed with other samples.

14. Centrifuge cells at 400× *g* for 5 min, discard the media, and add 1 mL of fresh DMEM-GHAA-5% FBS media. Seed the cells into 6-multiwell plates in 1 mL of total volume.

15. Three to five hours post seeding, change the media to 1.2 mL of DMEM-GHAA-5% FBS to remove dead cells and non-electroporated RNA transcripts.

16. Carefully wash cells after 24 h of post-electroporation with 1 mL of PBS.

17. Add 1.2 mL of VPSF-GHAA media and incubate electroporated cells for 24 h (MLB2) or 48–72 h (MLB1). Virus replication induces a cytopathic effect (CPE), which can be visualized under the microscope or quantified after fixing by staining nuclei and cells with Hoechst and WGA-FITC (Figure 2).

18. To ensure proper cell lysis, freeze the plate at -70 °C for at least 1 h, then thaw at room temperature. Collect both cells and the supernatant in the 1.5 mL microcentrifuge tubes.

19. Clarify viral supernatants by centrifugation at 7,000× *g* for 5 min, and then filter supernatants through a 0.2 μm filter.

20. Carefully transfer viral supernatants to a screw-cap tube and supplement with 5% glycerol and 0.2% BSA (final concentrations). These virus stocks are labeled as passage 0 (P0).

21. Generate P1, P2, etc., virus stocks by infecting cells with P0 virus using MOI 0.1 (Figure 3).

**Figure 3.**
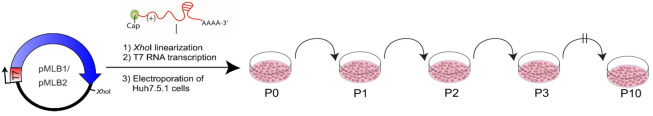
Method for a plasmid-derived reverse genetics (RG) system for MLB1 and MLB2. MLB cDNAs contain the entire genome flanked by the T7 promoter and XhoI linearization site. Huh7.5.1 cells were electroporated with full-genome T7 transcripts; the collected virus was used for serial passages (P1 to P10) in the same cell line (adapted from Ali et al. [1]).

**D. Titration of virus stocks using immunofluorescence-based detection**

1. Seed Huh7.5.1 cells (2.5–3 × 10^4^ cells per well) on 96-well flat-bottom plates 24 h before infection.

*Note: The cells should be 80%–90% confluent at the time of infection.*

2. Prepare 10-fold serial dilutions of virus stocks in the U-bottom 96-well plate. For 10-fold dilutions, use 135 μL of infection media and 15 μL of virus stock.

3. An example of a 96-well plate scheme used for titration of MLB1 virus stocks is given in Table 6.

**Table 6.** Preparation of virus dilutions

Dilution	1	2	3	4	5	6	7	8	9	10	11	12
10^-1^	MLB1 P1	MLB1 P2	MLB1 P3	MLB1 P4	MLB1 P5	MLB1 P6	MLB1 P7	MLB1 P8	MLB1 P9	MLB1 P10	Mock	MLB1 GNN
10^-2^	↓	↓	↓	↓	↓	↓	↓	↓	↓	↓	↓	↓
10^-3^	↓	↓	↓	↓	↓	↓	↓	↓	↓	↓	↓	↓
10^-4^	↓	↓	↓	↓	↓	↓	↓	↓	↓	↓	↓	↓

4. Discard the culture media from overnight cultured cells using a multichannel pipette or by inverting the plate against 5–10 layers of paper towels.

5. Infect monolayers of cells with 100 μL of diluted viruses.

*Note: Start from the lowest dilutions first, using the same multichannel pipette for the entire plate.*

6. Incubate the infected cells in the incubator (5% CO_2_, 37 °C) for 20–28 h (MLB2) and 40–48 h for MLB1.

7. Carefully remove the virus inoculum by tapping plates against 5–10 layers of paper towels, then remove paper towels into the autoclave bag. Alternatively, use a multichannel pipette to collect the virus-containing media into a plastic beaker containing 10% chloros to inactivate the virus in the safety cabinet. Follow local rules for the disposal of inactivated pathogens.

8. Fix the cells with 4% PFA in PBS (100 μL per well) for 20–30 min at room temperature. Disinfect the lid and borders of the 96-well plate with 4% PFA.

9. Before removing the plates from the safety cabinet, clean the outside of the plate with 1% Perasafe or 5% Chemgene.

10. Discard PFA after 30 min of fixing and wash the fixed cells with 1× PBS (100–200 μL per well). At this point, the plate can be stored at 4 °C or proceed directly for the immunostaining.

11. Permeabilize the fixed cells with 1% Triton X-100 (in 1× PBS) for 10 min on the rocker platform.

12. Wash the cells twice with 200 μL of 1× PBS.

13. Add 45 μL of diluted primary antibody per well to the cells and incubate on a rocker platform for 1–2 h at room temperature or 4 °C overnight. For MLB1/MLB2 virus capsid protein detection, use anti-MLB1 CP antibody (rabbit polyclonal, in-house, 1:300 is diluted in antibody solution). This antibody can be substituted with another custom-generated antibody against capsid-derived peptides.

14. Wash the cells three times with 200 μL of 1×PBS per well and incubate on the rocker for 5 min during each wash.

15. Add 45 μL of diluted secondary antibody (1:3,000) per well and incubate the plate on a shaker for 1–2 h at room temperature. Prepare anti-rabbit IRDye 800 secondary antibody in the antibody solution.

16. Discard the secondary antibody and wash cells three times with 200 μL of PBS per well; incubate on the rocker for 5 min during each wash.

17. Add 100 μL of PBS per well and clean the bottom of the plate with 70% ethanol.

18. Scan the plate on the LICOR imager using plate settings (+3 mm, medium, 84 μm). Each scan takes 25–30 min.

19. Export a 300 dpi image and quantify individual capsid-positive cells in the lowest dilution. Calculate the virus titer, taking the dilution factor into account. Using this method, titers of astroviruses are determined as infectious units per milliliter (IU/mL). An example of the MLB1 virus titration is shown in Figure 4.

**Figure 4.**
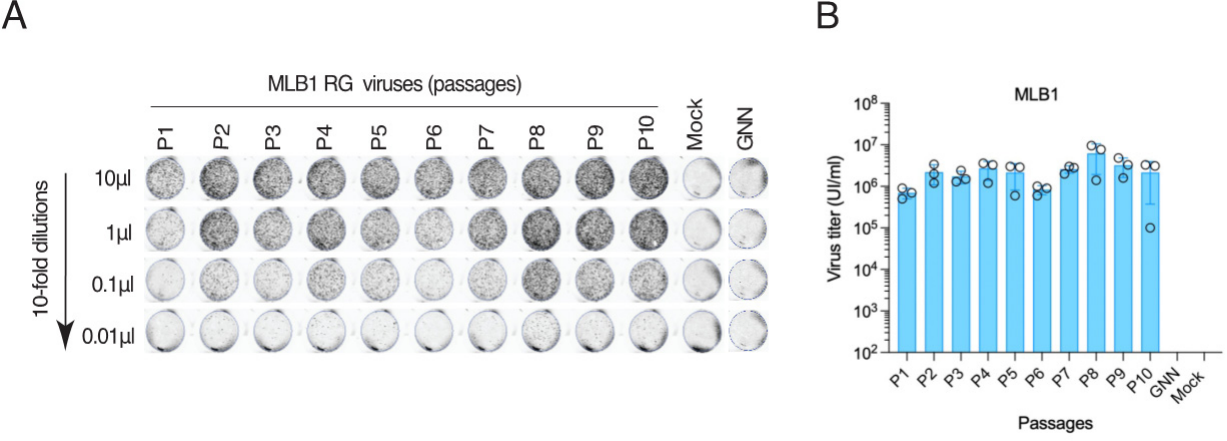
Titration of MLB1 RG viruses. A. Huh7.5.1 cells are seeded on a 96-well plate, infected with 10-fold serial dilutions of MLB1 and fixed at 40–48 hpi, permeabilized, stained with anti-MLB1 capsid antibody, and imaged by Licor imager. B. Graph showing the titers of MLB1 viruses of 10 serial passages (P1 to P10). The MLB1 GNN mutant (GDD→GNN) harbors point mutations in the active site (GDD) of the viral RNA-dependent RNA polymerase (RdRp). Infectious units/mL = (No. of capsid-positive signals/0.01) × 1,000 μL/mL. Adapted from Ali et al. [1]. Data are mean ± SEM of 3 independent experiments.

**Part II: Measuring MLB RG virus replication kinetics by western blotting**

1. Seed Huh7.5.1 cells in the 6-multiwell plate (2.5 × 10^5^/well) in 2 mL of complete media 24 h before infection.

*Note: This should result in 60%–75% confluent cells.*

2. Wash cells with 1 mL of DMEM-GHAA media.

3. Infect cells with MOI 1 of MLB1 and MLB2 viruses in a total volume of 250 μL of DMEM-GHAA media.

4. Incubate the cells for 1–2 h on a rocker at room temperature.

5. Add 1 mL of DMEM-GHAA-5% FBS media and carefully transfer the infected cells into the incubator (37 °C with 5% CO_2_).

6. Collect the infected cells at 24, 48, 72, and 96 h post infection. Since CPE can be present throughout the experiment, collect both media and cells by pipetting or scraping into a 1.5 mL Eppendorf tube, followed by centrifugation at 2,000× *g* for 5 min at 4 °C.

7. Discard the supernatants and resuspend the cell pellet in 80 μL of 50 mM Tris HCl pH 6.8, followed by the addition of 85 μL 2× LB-βME.

8. Immediately denature cell lysates by boiling at 95 °C for 5 min, followed by a spin for 1 min at 9,600× *g*.

9. Load 10 μL of cell lysates from each time point onto an 8%–12% SDS-PAGE gel and run the PAGE using standard running conditions (120 V for 1 h).

10. Transfer the resolved proteins to 0.2 μm nitrocellulose membranes using semi-dry transfer with a standard protocol (25 V, 1 A for 30 min).

11. Block the membrane with 4% milk in PBS for 1 h at room temperature.

12. Incubate the membrane with anti-MLB1 capsid antibody (rabbit polyclonal antibody, 1:3,000) and anti-tubulin (Abcam, ab6160, 1:1,000) for 1 h at room temperature or overnight at 4 °C on a rocker.

13. Wash the membrane three times with 0.1% PBST on a rocker for 5 min each time.

14. Add secondary antibodies, anti-rabbit (LI-COR IRDye 800, 1:3,000) and anti-mouse (LI-COR IRDye 680, 1:3,000), to the membrane and incubate for 1 h at room temperature or overnight at 4 °C on a rocker.

15. Wash the membrane three times with 0.1% PBST on a rocker for 5 min each time.

16. Scan immunoblot on a LI-COR ODYSSEY CLx imager and analyze using Image Studio version 5.2 (an example is shown in Figure 5).

**Figure 5.**
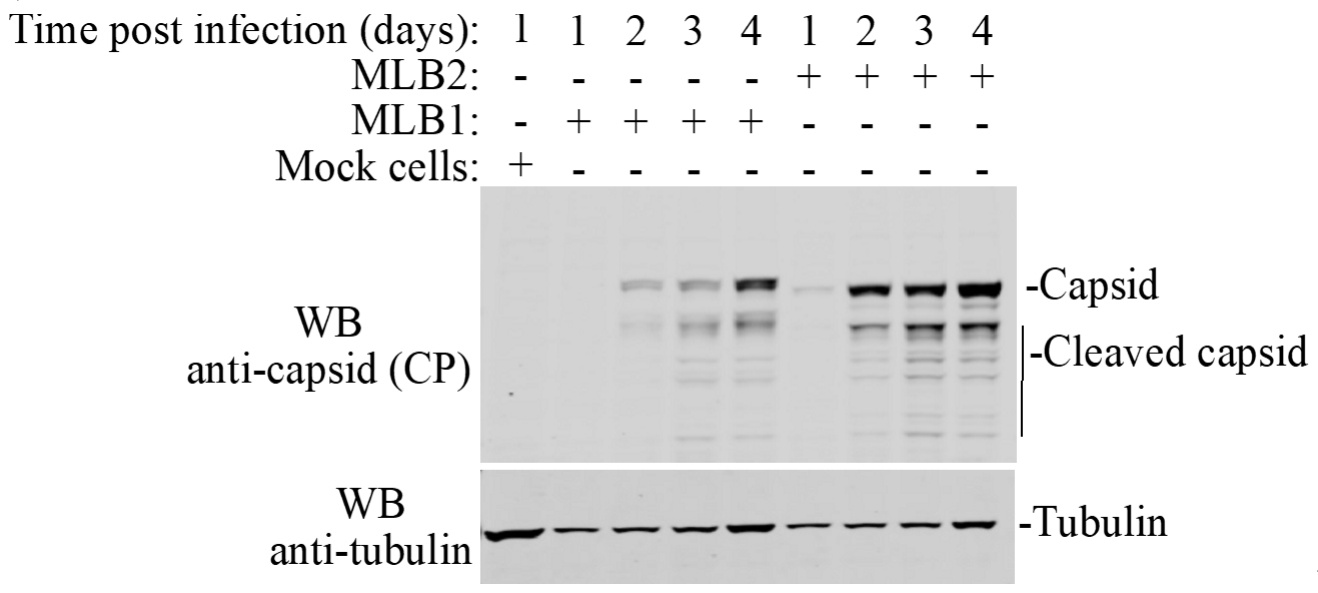
Huh7.5.1 cells are infected with MLB1 and MLB2 viruses with MOI 1, viral capsid proteins were analyzed at the indicated time points using anti-CP MLB1 antibody (MLB1 CP antibody also detects MLB2 capsid protein). Anti-tubulin antibody is used as a loading control. This variation in tubulin levels can be explained by the combination of cell growth and virus-induced cell death and can be included in quantification if necessary.

**Part III: Detection of virus infection by immunofluorescence**

1. Day 1: Seed 5 × 10^4^ Huh7.5.1 cells on IBIDI 8-well chambered slides (or grown on the sterile coverslips in a 12-multiwell plate at 1 × 10^5^ cells per well).

2. Day 2: To infect the cells, first wash them with 1 mL of DMEM-GHAA media, and then infect with MOI 0.1 of MLB2 (or MLB1) virus in a total volume of 250 μL of DMEM-GHAA (keep one well of mock cells as a negative control).

3. Incubate cells for 1–2 h on a rocker at room temperature.

4. Remove virus inoculum and add 300 μL of fresh DMEM-GHAA-5% FBS media to each well. Then, carefully transfer infected cells to the incubator (37 °C with 5% CO_2_).

5. Fix the infected cells at 24, 48 and 72 h post infection. First, wash cells with 1× PBS, and then fix them with 10% formaldehyde in PHEM buffer (1:1) for 15 min. Alternatively, the cells can be fixed with 4% PFA in PBS.

6. Wash fixed cells three times with 1× PBS. At this stage, the fixed cells can be stored at 4 °C in PBS or directly proceed for immunofluorescence.

7. Permeabilize cells with 0.1% Triton X-100 for 10 min at room temperature.

8. Wash the cells three times with 1× PBS.

9. Block the cells with 2% goat serum in 1× PBS for 1 h at room temperature.

10. Add diluted primary antibodies (MLB1 anti-capsid (1:300) and anti-dsRNA IgG2a (Scicons J2, 1:250) to the cells and incubate them for 1 h at room temperature or overnight at 4 °C.

11. Wash the cells three times with 1× PBS to remove unbound antibodies.

12. Add diluted secondary antibodies (anti-rabbit Alexa 488- or anti-mouse 594-conjugated secondary antibody, 1:1,000) to the cells and incubate for 1 h at room temperature or overnight at 4 °C.

13. Wash the cells three times with 1× PBS to remove unbound antibodies.

14. Add 1:12,000 diluted Hoechst (in 1× PBS) to counterstain nuclei for 5 min at room temperature. Replace with 1× PBS.

15. Image MLB2 virus-infected cells using a Leica SP5 confocal microscope with a water-immersion 63Å objective or any similar confocal imaging platform.

**Figure 6.**
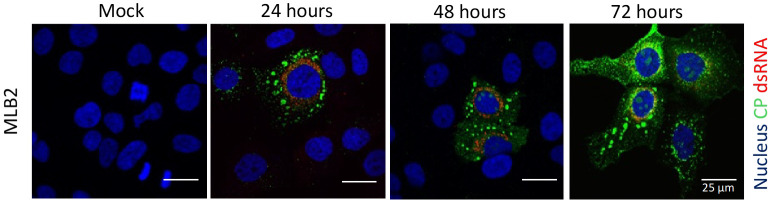
Representative confocal images of MLB2-infected Huh7.5.1 cells. MLB2 virus-infected cells are positive for capsid viral protein (in green), and virus replication is detected by using anti-dsRNA antibody (in red). Adapted from Ali et al. [2].

An example of MLB2 virus infection/replication detection using this method is shown in Figure 6.

**Part IV. Construction of MLB replicons and replicon assay**

Replicon systems are considered valuable tools for the rapid measurement of RNA replication by inserting either fluorescent or luminescent reporter genes in place of the structural proteins coding sequence without altering the regulatory RNA elements essential for virus replication.

**A. Engineering of MLB replicons**

**Figure 7.**
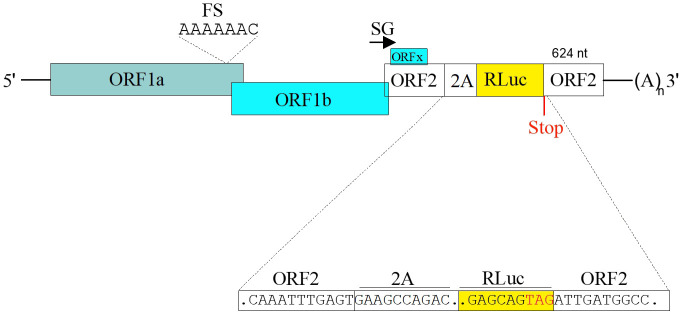
Schematic of the MLB replicons. The 2A-RLuc cassette is fused in the ORF2 followed by the stop codon and extended 3′ UTR. SG indicates “subgenomic promoter.” Adapted from Ali et al. [1].

1. To generate MLB1 and MLB2 replicon systems, both the MLB1 (GenBank accession number ON398705) and MLB2 (GenBank accession number ON398706) RG clones are kept intact up to the end of ORFX, followed by a foot-and-mouth disease virus 2A sequence and a Renilla luciferase (Rluc) sequence with a stop codon, followed by the last 624 nucleotides of the virus genomes and a 35-nucleotide poly-A tail (Figure 7).

**B. In vitro synthesis of replicon T7 RNA transcripts**

1. Linearize replicon-containing plasmids (pMLB1R and pMLB2R, pMLB1R-GNN and pMLB2R-GNN) by digesting with the XhoI restriction enzyme.

2. Purify linearized plasmids using the PCR Clean-Up kit and elute in 20 μL of MilliQ water or elution buffer. Quantify concentrations of linearized plasmids using a Nanodrop.

3. Synthesize capped T7 RNA transcripts using T7 mMESSAGE mMACHINE Transcription kit (as shown in Table 7).

**Table 7.** In vitro synthesis of replicon T7 RNA transcripts

	Components	Volumes
1	Linear plasmids (100–200 ng)	1.5 μL
2	2× NTP/CAP	2.5 μL
3	10× transcription buffer	0.5 μL
4	T7 Enzyme mixture	0.5 μL
Total reaction volume		5.0 μL

**Figure 8.**
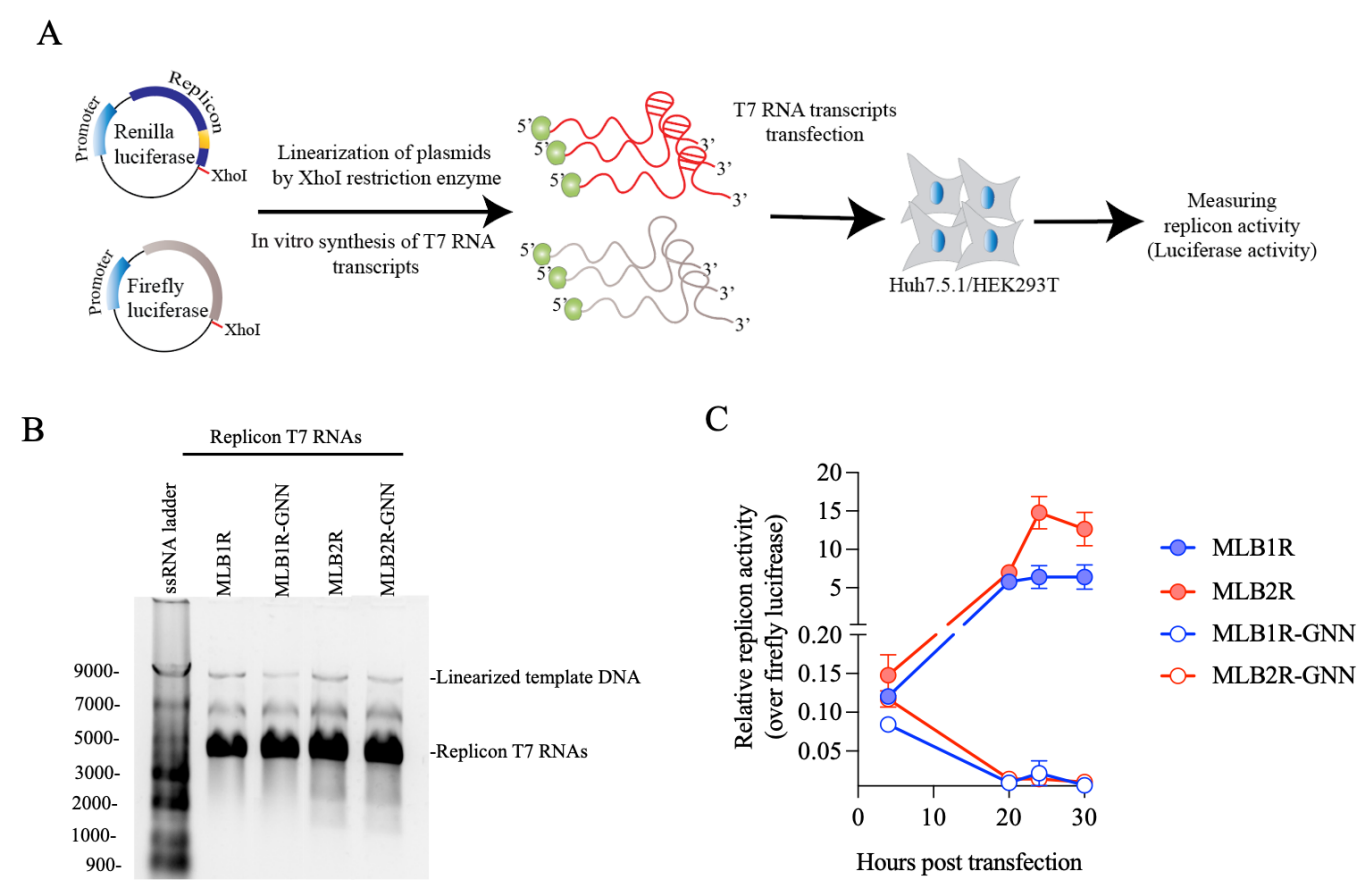
Strategy to rapidly assess the replication dynamics of MLB astroviruses. A. Plasmids containing MLB replicon with Renilla luciferase and Firefly luciferase under the T7 promoter are linearized and used as a template for in vitro T7 RNA synthesis, followed by transfection and measurement of luciferase activity. B. The T7 RNA transcripts are checked on the 1% agarose gel. C. MLB1 and MLB2 replicon activities (replication dynamics) are measured in Huh7.5.1 cells. The replicon activity is normalized over firefly luciferase and shown as a relative replicon activity. Data are mean ± SD of 3 independent experiments. A RdRp knockout mutant (GDD→GNN) is used as replication-deficient control.

4. Incubate the T7 RNA synthesis reactions at 37 °C for 60–90 min.

5. Add DNase I (2 U) for 20 min at room temperature to remove template plasmid DNA.

6. Check the T7-transcribed RNA(s) integrity by running on 1% agarose gel before or after DNase I digestion (Figure 8B).

7. Purify the T7-transcribed RNA(s) using an RNA Clean-Up and Concentrator kit and quantify concentrations using a Nanodrop, adjusting RNA concentration to 100–200 ng/μL.

**C. Transfection of replicon T7-transcribed RNA(s)**

1. Grow the Huh7.5.1 cells until they reach 70%–90% confluency (a T175 flask is sufficient for two 96-well plates).

2. Wash the cells with 10 mL of 1× PBS.

3. Trypsinize cells using 2–3 mL of 0.08% trypsin-EDTA at room temperature for 2–3 min.

4. Add 10 mL of DMEM-GH-2% FBS to neutralize trypsin activity and collect cells into 50 mL Falcon tubes.

5. Centrifuge the cells at 400× *g* for 5 min (with no brake, centrifugation takes more time to stop; so, in the meantime, start preparing transfection mixtures).

6. Discard the media and resuspend cells in 25 mL of DMEM-GH media.

7. Centrifuge the cells again at 400× *g* for 5 min (with no brake).

8. Discard the media and resuspend the cells in fresh DMEM-GH media to obtain a concentration of 1 × 10^6^ cells/mL (at this point, cells are ready for transfection, so it is recommended to proceed immediately).

9. Prepare the transfection mixtures in the transfection media [serum-free OptiMEM containing RNaseOUT (40 units/mL)]. For mix 1, add (number of samples + 10%) × 0.5 μL of Lipofectamine 2000 and (number of samples + 10%) × 0.5 μL of OptiMEM in a 1.5 mL tube, mix well, and incubate for 5 min at room temperature. For mix 2, add 100 ng of T7-transcribed RNA(s) [90 ng of replicon T7-transcribed RNA(s) + 10 ng of T7-transcribed RNA(s), Renilla firefly internal control] in 10 μL of OptiMEM. For internal control, Firefly luciferase gene cloned under T7 promoter (Promega or similar) is used (cap-dependent translation should be consistent between replicates/samples).

10. After 5 min, combine mixes 1 and 2 and incubate them for 20 min at room temperature.

11. Add 100 μL of the prewashed cells (10^5^ cells per well) to the transfection mixture and incubate at room temperature for 2–3 min.

12. Add FBS (5% final concentration) and then quickly transfer the cells into a flat-bottom 96-well plate.

13. Incubate transfected cells in a 37 °C incubator with 5% CO_2_.

14. Discard the media from cells at 4, 20, 24, and 30 h post-transfection and lyse the cells in the freshly prepared 1× passive lysis buffer (100 μL per well). To ensure complete cell lysis, freeze the plates at -70 °C for at least 30 min.

15. Thaw the plates at room temperature for 30 min and measure luciferase activity.

**D. Measuring replicon activity by luciferase assay**

1. To measure the luciferase reporter activity, take 20 μL of cell lysates and add 20 μL of Luciferase Assay Reagent II, followed by measurement of Firefly luciferase activity using GloMax^®^ Navigator microplate luminometer.

2. Then, add 20 μL of freshly made 1× Stop & Glo reagent and incubate away from light for 10 min, followed by measurement of Renilla luciferase activity.

3. Calculate replicon activity as the ratio of Renilla luciferase (subgenomic reporter) to Firefly luciferase [co-transfected control T7-transcribed RNA(s)]. Subtract the background (mock-transfected cells) from each reading. The activity of the GNN mutant is used as a negative control, indicating no replication but active initial translation. The pMLB1R-GNN and pMLB2R-GNN (GDD→GNN) mutants carry point mutations in the active site (GDD) of the viral RNA-dependent RNA polymerase (RdRp), an enzyme critical for the replication of viral genomes.

An example of MLB1 and MLB2 replicon assay is shown in Figure 8.

## Data analysis

Data are shown as mean ± SEM (n = 3 independent experiments).

Data are shown as mean ± SD (n = 3 independent experiments).

## Validation of protocol

The reproducibility of the protocol has been described in the following research articles:

• Ali et al. [1]. Attenuation hotspots in neurotropic human astroviruses. *Plos Biology*.

• Ali et al. [2] The astrovirus N-terminal nonstructural protein anchors replication complexes to the perinuclear ER membranes. *Plos Pathogens*.

## General notes and troubleshooting

**Troubleshooting**

Ensure that RNA quality is high, as poor quality can negatively affect transfection/electroporation efficiency.

Confirm the sequence of reverse genetics and replicon plasmids before using them in the experiments.
